# Periostin level in gingival crevicular fluid in periodontal disease: a systematic review and meta-analysis

**DOI:** 10.1186/s12903-023-03031-w

**Published:** 2023-05-12

**Authors:** Fatemeh Abdolalian, Mojtaba Bayani, Saeid Afzali, Afrooz Nakhostin, Amir Almasi-Hashiani

**Affiliations:** 1 Department of Periodontics, School of Dentistry, Arak University of Medical Sciences, Arak, Iran; 2 Student Research Committee, Arak University of Medical Sciences, Arak, Iran; 3 Department of Restorative Dentistry, School of Dentistry, Arak University of Medical Sciences, Arak, Iran; 4 Department of Epidemiology, School of Health, Arak University of Medical Sciences, Arak, Iran; 5 Traditional and Complementary Medicine Research Center (TCMRC), Arak University of Medical Sciences, Arak, Iran

**Keywords:** Periostin, Periodontal disease, Gingival Crevicular fluid, Systematic review, Meta-analysis

## Abstract

**Background:**

Periostin, a secreted adhesion molecule, is a matricellular protein secreted most in periodontal ligament and periosteum. Periostin is also needed for integrity and maturation of periodontal tissue. This meta-analysis was conducted to compare the gingival crevicular fluid (GCF) periostin levels in subjects having periodontal disease and healthy periodontium.

**Methods:**

In this meta-analysis, three international database including PubMed, Scopus and Web of Science were searched and 207 studies retrieved. Also, the Google Scholar was searched to find more related studies (two studies were found). To assess the risk of bias of included studies, the Newcastle–Ottawa assessment scale adapted for case–control was used. Finally, required data was extracted and included into analysis. All statistical analysis were done using Stata software.

**Results:**

Eight studies were included in this meta-analysis. Results showed that GCF periostin level is significant lower in chronic periodontitis group compare to healthy people (the standardized mean difference (SMD) = -3.15, 95% CI = -4.45, -1.85, *p* < 0.001). The syntheses of studies shown a significant decrease in the periostin level of chronic periodontitis patients compared to the gingivitis patients (SMD = -1.50, 95%CI = -2.52, -0.49, *P* = 0.003), while the mean level of periostin between the gingivitis patients and healthy group has no significant difference (SMD = -0.88, 95%CI = -2.14, 0.38, P = 0.173).

**Conclusion:**

The mean concentration of GCF periostin in people with chronic periodontitis significantly decreased compared to people with gingivitis and also compared to healthy people, while no significant difference was observed between the two groups with gingivitis and healthy people. Therefore, this marker may be used as a diagnostic criterion for the disease, which requires further studies.

## Introduction

Periodontium is the supporting structure of teeth, which includes periodontal ligament, cementum, gum and alveolar bone [1]. The main task of this tissue is to connect the tooth to the bone and create a barrier against the microflora of the mouth to protect the underlying structures [2]. If this tissue is exposed to pathological conditions, periodontal disease occurs. This disease is a set of biological processes that include interactions between microorganisms and the immune-inflammatory responses [3]. It is also thought that the imbalance in the immune-inflammatory responses creates the background of the pathogenesis of inflammatory diseases of the periodontium, such as periodontitis [4]. Periodontal destruction in periodontal diseases is caused by local pro-inflammatory cytokines produced in response to bacterial infection [5].

Gingival crevicular fluid (GCF) is a liquid containing a complex combination of substances that is released in the gingival crevice, a space between the tooth and the non-adherent gum. GCF, which originates from the gingival vascular network, flows through the outer basement membrane and junctional epithelium to reach the gingival groove. This liquid is derived from serum, host inflammatory cells, periodontal structural cells and oral bacteria [6, 7]. Also, GCF contains substances such as substances resulting from tissue destruction, inflammatory mediators and antibodies against dental plaque bacteria [8]. Sampling of GCF can be used as one of the least invasive (nontraumatic) and most valuable diagnostic methods that provide information about the health of periodontal tissue, including the condition of connective tissue and the degree of destruction of hard tissue [9].

Periostin (POSTN for short) is an 811-amino-acid protein that acts as a matricellular protein (a non-structural protein in the intercellular matrix) in cell activation by binding to cell surface receptors and plays various roles such as cell migration, wound healing, tooth growth, etc. [10–12]. Periostin is known to be important in regulating bone formation [13]. Also, biologically, periostin provides stable conditions for maintaining the integrity of connective tissue [14]. This protein is called periostin due to its expression in periosteum and periodontal ligaments [15].

Considering the high prevalence of periodontal diseases worldwide and the destructive and irreversible effects of this disease on oral health and quality of life and the importance of early diagnosis, therefore, periostin can be used as a possible biomarker for the early diagnosis of this disease. Considering the number of studies conducted in this field and the lack of a comprehensive conclusion, we aimed to conduct a comprehensive systematic review and meta-analysis regarding the possible role of GCF periostin as a new potential biomarker in the diagnosis of periodontal diseases.

## Methods

### Study design

This systematic review and meta-analysis was conducted based on PRISMA guideline (Preferred Reporting Items for Systematic Reviews and Meta-Analyses) [16]. This study has been approved by the ethics committee of Arak University of Medical Sciences with the ethics code IR.ARAKMU.REC.1400.013.

### Eligibility criteria

The inclusion criteria included the following: studies that examined GCF periostin levels in samples from healthy subjects (control group) and subjects with periodontal disease, studies that included clinical periodontal assessment including periodontal pocket depth (PPD) and measurement of clinical attachment loss (CAL), cross-sectional, case control and cohort studies, as well as studies that had English full-text and were published in journals and congresses. There was no publication date limit. The exclusion criteria included the following. Studies that were in vitro and animal studies, studies that did not have relevant data, and studies that did not have access to the English full text. Periodontitis, gingivitis and periodontal health classified based on 1999 Periodontal Disease Classification.

### Information sources

Three international databases were searched. Medline (via PubMed), Web of science and Scopus databases were searched according to the search query and keywords to identify relevant studies until March 15 2023. In addition, Google Scholar was used to find the gray literature and also, the references list of included studies were searched manually.

### Search strategy

Various keywords were used for searching in the mentioned three international databases. As an example, the keywords used in the PubMed are “Periodontal Diseases”, “Periodontitis”, “Gingivitis”, “Periodontal Index”, “Periostin”, “POSTN”, “Osteoblast-specific factor”, “OSF-2 protein”, “Gingival Crevicular Fluid” and “GCF”. The searches were done by AAH. The search strategy in Medline is reported in Table 1. The tags of medical subject heading (MeSH) and text word (TW) was used to precise search. The searches were done by AAH.

**Table 1:** The details of search strategy in Medline

Search	Query	Results
#3	Search: ("POSTN protein, human" [Supplementary Concept] OR "Periostin" [tw] OR "Osteoblast-specific factor 2"[tw] OR "OSF-2 protein"[tw]) AND ("Periodontal Diseases"[Mesh] OR "Periodontal Diseas*"[tw] OR "Periodontitis"[Mesh] OR "Periodontitis"[tw] OR "Chronic Periodontitis"[Mesh] OR "Chronic Periodontitis"[tw] OR "gingivitis"[Mesh] OR Gingivitis[tw] OR "periodontal index"[Mesh] OR Periodontal Index[tw] OR "aggressive periodontitis"[Mesh] OR "Aggressive Periodontitis"[tw] OR "periodontal inflammation"[tw]) Sort by: Most Recent	65
#2	Search: "POSTN protein, human" [Supplementary Concept] OR "Periostin" [tw] OR "Osteoblast-specific factor 2"[tw] OR "OSF-2 protein"[tw] Sort by: Most Recent	2259
#1	Search: "Periodontal Diseases"[Mesh] OR "Periodontal Diseas*"[tw] OR "Periodontitis"[Mesh] OR "Periodontitis"[tw] OR "Chronic Periodontitis"[Mesh] OR "Chronic Periodontitis"[tw] OR "gingivitis"[Mesh] OR Gingivitis[tw] OR "periodontal index"[Mesh] OR Periodontal Index[tw] OR "aggressive periodontitis"[Mesh] OR "Aggressive Periodontitis"[tw] OR "periodontal inflammation"[tw] Sort by: Most Recent	113,948

### Selection process

After searching and finding the related studies in the databases, the studies were entered into the Endnote software, and at first the duplicate articles were removed, then the remaining articles were screened in terms of title and abstract, and related, duplicate and suspicious studies were kept, and unrelated articles were removed. In the next step, the full-text of the remaining articles was screened and irrelevant studies were removed. Finally, related studies were reviewed to check the inclusion and exclusion criteria, and unrelated articles were discarded, and suitable studies that had the desired data of this study were included in the meta-analysis. The studies were screened by SA under the supervising of MB and AAH.

### Data collection process and data items

Data were extracted by SA. An email was sent to the authors of studies whose full-text was not available or required numerical data was not reported in the paper. From the all included studies, the last name of first author, the publication date, country, the sample size of cases with periodontal disease and control group, the mean age of the participants, the method of measuring the GCF periostin level, study design, the mean and standard deviation of the GCF periostin level in both groups, and the quality score on each paper were extracted in recorded in Excel software.

### Study risk of bias assessment

The risk of bias of the included articles in the meta-analysis was checked by the standard Newcastle–Ottawa assessment checklist [17], which is designed for observational studies including cross-sectional, case–control, and cohort studies. This checklist gives the articles a score of 0 to 9, and finally the articles are placed in three categories with low quality (score 0 to 3), medium quality (score 3 to 6) and high quality (score 6 to 9).

### Effect measures

In this study, our aim was to compare the mean level of periostin between two groups including periodontitis patients and healthy people. Therefore, the mean difference was used as effect measures and finally the standardized mean difference (SMD) and its 95% confidence interval (VI) is used as a summary statistic in this meta-analysis.

### Statistical analysis

Chi-square test and I-square statistic were used to evaluate the heterogeneity between included studies, and I-square above 50% was measured as significant heterogeneity and random-effects model was used to pool the data when there was significant heterogeneity, and also in cases where there was no significant heterogeneity, fixed-effects model was used to calculate SMD and its 95% confidence limits. To check the existence of publication bias, the graphical method of funnel plot and also Begg test were used. All statistical analysis were conducted using Stata software version 16 (Stata Corp, College Station, TX, USA).

## Results

### Study selection

**Fig. 1:**
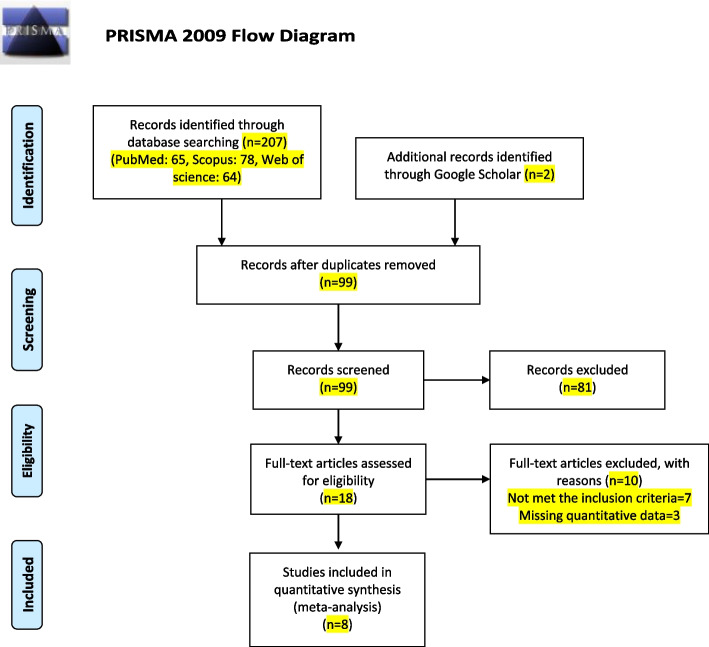
Flow diagram of the literature search for studies included in meta-analysis

According to the search strategy, 209 articles were identified. Out of 209 articles, 65 articles were the result of searching in PubMed databases, 78 studies were identified in Scopus, and 64 articles were found from Web of Science database. Also, in the review of gray literatures, 200 articles (20 pages) from google scholar were reviewed by manual search, and the title and abstract of the articles and their full-text were checked if needed, and two papers retrieved. After removing duplicates, 99 articles remained for title and abstract screening. After screening the title and abstract of the remaining articles, 81 irrelevant articles were removed. The full-text of eighteen remaining studies were screened and ten unrelated studies which not meet the inclusion criteria were excluded. Finally, eight eligible studies [11, 18–24] were included in the meta-analysis. The more details on study process were presented in Fig. 1.

### Study characteristics

All the included studies were case–control study and totally, 486 cases (197 healthy people, 197 chronic periodontitis and 92 Gingivitis) were included in the meta-analysis. The oldest study was conducted in 2014 [19] and the newest in 2022 [24]. In all studies. ELISA method was used to assess the GCF periostin level and the unit of measurement was converted to ng/ml in all studies. More details about the articles included in the meta-analysis are reported in Table 2. In addition, details of the studies regarding GCF sampling, method of storing the samples, ELISA kits, laboratory analysis are reported in Table 3.

**Table 2:** A summary of the included studies characteristics in the meta-analysis

Author	Country	Sample size of healthy group	Sample size of chronic periodontitis	Sample size of gingivitis	Periostin mean in healthy group	Periostin mean in chronic periodontitis	Periostin mean in gingivitis	Mean age of healthy group	Mean age of chronic periodontitis	Mean age of gingivitis	Quality score
Balli U (2014), [19]	Turkey	20	30	30	346.93 (118.22)	51.64 (15.99)	108.86 (34.88)	37.75	41.17	38.27	7
Jamesha FI (2018), [20]	India	13	13	-	182.41 (35.5)	79.86 (25.91)	-	24.69	43.46	-	7
Sophia K (2020), [11]	India	30	30	30	24.77 (4.46)	15.86 (2.85)	21.46 (2.97)	-	-	-	5
Radhika BN (2019), [22]	India	20	20	-	27.52 (2.39)	20.18 (1.42)	-	36.39	36.39	-	8
Momeni F (2020), [21]	Iran	53	53	-	0.0297 (0.00459)	0.0101 (0.0016)	-	39.98	40.24	-	7
Rezaei B (2021), [23]	Iran	29	29	-	0.0098 (0.0031)	0.0037 (0.001)	-	46.27	45.11	-	8
Akman AC (2018), [18]	Turkey	10	-	10	11.29 (15.38)	-	16.15 (17.9)	43.23	-	43.23	7
Sari A (2022), [24]	Turkey	22	22	22	29.3 (22.7)	19 (14.1)	30.3 (29)	33	39.5	32.5	8

**Table 3:** A summary of GCF sampling, method of storing the samples, ELISA kits and laboratory analysis of the included studies

Author	GCF sampling	Method of storing the sample	laboratory analysis	ELISA Kit
Balli U (2014), [19]	Gingival crevicular fluid was sampled with filter paper using the intracrevicular method	The samples were placed into a sterile polypropylene tube and stored at -70 °C until analysis	ELISA Kit	Commercially available Periostin kit (Uscn Life Science Inc., Wuhan, China)
Jamesha FI (2018), [20]	Microcapillary pipettes	Wrapped in aluminum foil, placed inside separate sterile tubes and stored at − 80 °C in an ultra‑low temperature freezer	ELISA Kit	–
Sophia K (2020), [11]	Microcapillary pipettes	The GCF and serum samples were stored at -70 °C until the time of assay	ELISA Kit	–
Radhika BN (2019), [22]	Microcapillary pipette	Eppendorf tube and stored at –80 °C in a deep freezer, until further processing	ELISA Kit	Bioassay Technology Laboratory, 1713, Junjiang International Bldg, Yangpu Dist, Shanghai, China
Momeni F (2020), [21]	Paper strips	Store in buffer solution tube at -20 c	ELISA Kit	–
Rezaei B (2021), [23]	Paper strips	Placed into microcentrifuge tubes and stored at − 20 °C	ELISA Kit	–
Akman AC (2018), [18]	Standardized paper strips were inserted 1 mm depth	GCF samples were placed in sterile Eppendorf tubes and carefully wrapped to be stored in -80 °C	ELISA Kit	Human periostin ELISA kit Periostin (CSB-E16444h) Cusabio, Wuhan, China
Sari A (2022), [24]	The samples were collected within 30 s with standardized paper strips (Periopaper; Orafow Inc., Plainview, NY) by the orifce method	Periopaper strips were pooled in plastic Eppendorf microcentrifuge tubes and stored at – 80 °C	ELISA Kit	Elabscience, catalog no: E-EL-H6113

### Risk of bias in studies

Checking the quality of studies using the Newcastle–Ottawa Scale checklist is shown in Table 2. The quality of one study (12.5%) was medium and seven studies (87.5%) were high.

### Results of syntheses, heterogeneity and additional analysis

#### Comparison of periostin level in GCF of chronic periodontitis patients and healthy individuals

**Fig. 2:**
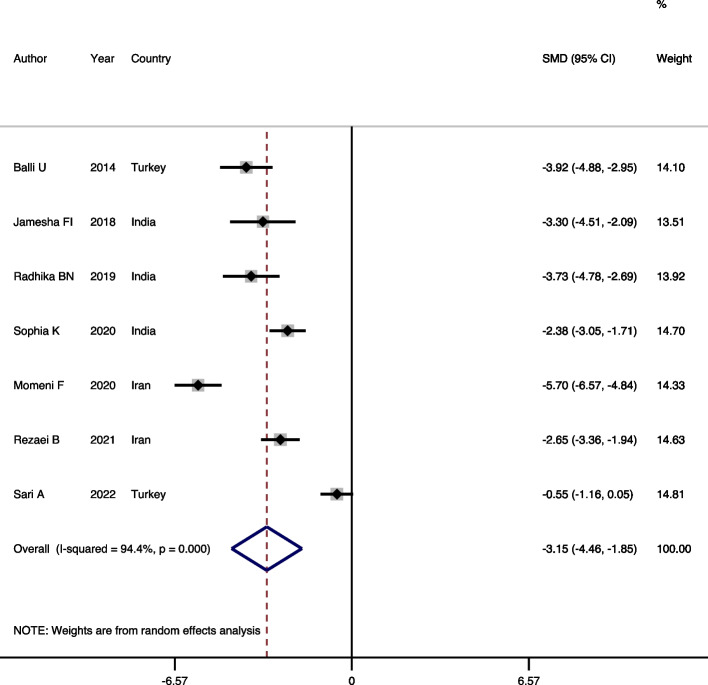
Forest plot comparing the GCF periostin level of chronic periodontitis patients and healthy individuals

Seven studies [11, 19–24] were included in this comparison. The results of assessing heterogeneity among studies suggested a significant heterogeneity (Heterogeneity chi-squared = 106.8, d.f = 6, *p* < 0.001, I-squared (variation in SMD attributable to heterogeneity) = 94.4%, estimate of between-study variance Tau-squared = 2.88)) and the random-effects model was used to poll the results. The syntheses of studies revealed a significant decrease in the periostin level of chronic periodontitis patients compared to the healthy individuals (SMD = -3.15, 95% CI = -4.45, -1.85, *p* < 0.001) (Fig. 2). The symmetrical distribution of funnel plot, and also the results of Begg test (*p* = 0.230) suggested no evidences in favor of publication bias.

#### Comparison of periostin level in GCF of chronic periodontitis and gingivitis patients

**Fig. 3:**
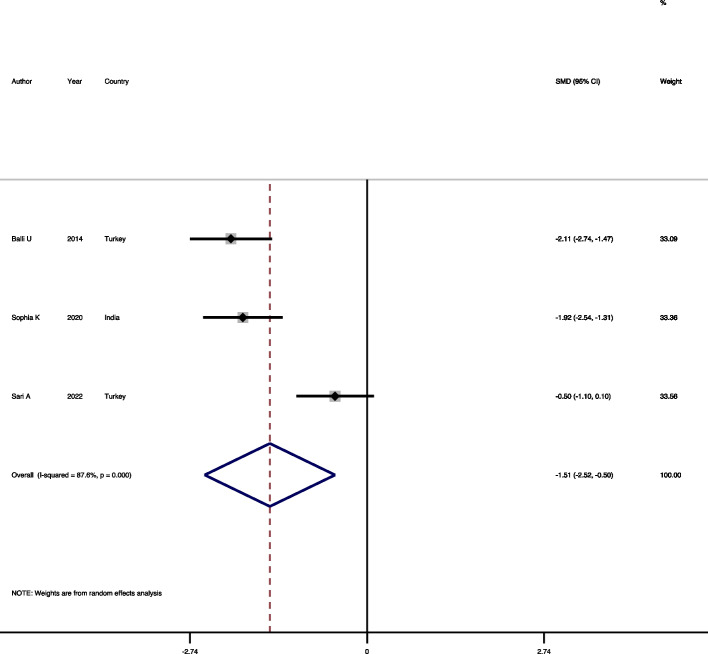
Forest plot comparing the GCF periostin level of chronic periodontitis patients and gingivitis patients

In three studies [11, 19, 24], the mean of periostin level in GCF of chronic periodontitis and gingivitis patients were compared and included in this analysis. The results showed that there is a significant heterogeneity among studies and random-effects model was used to poll the results (Heterogeneity chi-squared = 16.1, d.f. = 2, *p* = 0.001, I-squared = 87.6%, estimate of between-study variance Tau-squared = 0.69). The syntheses of studies shown a significant decrease in the periostin level of chronic periodontitis patients compared to the gingivitis patients (SMD = -1.50, 95%CI = -2.52, -0.49, *P* = 0.003) (Fig. 3). Regarding publication bias, the funnel plot and Begg test (*p* = 0.296) suggested no evidences in support of publication bias.

#### Comparison of periostin level in GCF of gingivitis patients and healthy individuals

**Fig. 4:**
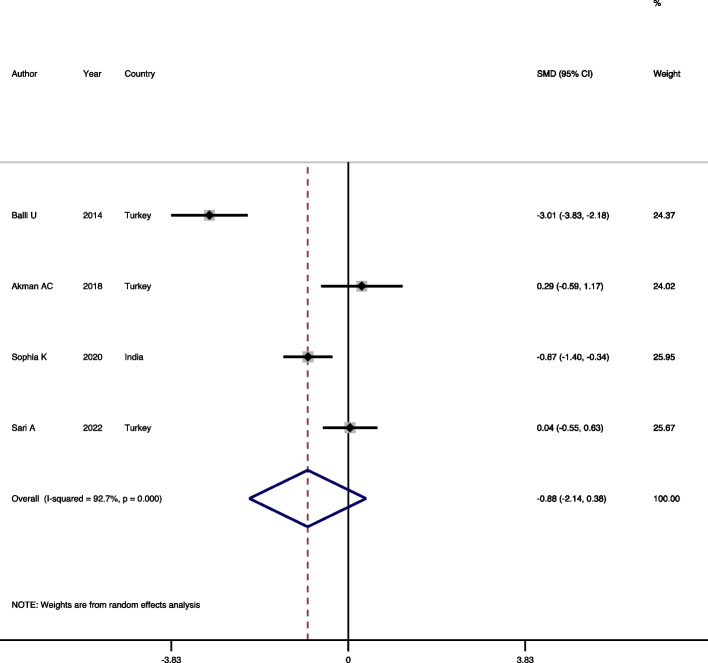
Forest plot comparing the GCF periostin level in gingivitis patients and healthy individuals

To compare the mean concentration of periostin between two groups of gingivitis and healthy individuals, four studies [11, 18, 19, 24] were included in the meta-analysis. Examining the results of the existence of heterogeneity showed that there is a significant heterogeneity between the studies, therefore, the Random-effects model was used to combine the findings of the studies (heterogeneity chi-squared = 41.1, d.f. = 3, *p* = 0.001, I-squared = 92.7%, estimate of between-study variance Tau-squared = 1.52). The results of this meta-analysis showed that the mean level of periostin between the two mentioned groups has no significant difference (SMD = -0.88, 95%CI = -2.14, 0.38, *P* = 0.173) (Fig. 4). Regarding publication bias, the funnel plot and Begg test (*p* = 0.999) suggested that there are no evidences in favor of publication bias.

## Discussion

This review study examined the findings of studies related to the examination of periostin levels in people with chronic periodontitis and comparing it with healthy people and people with gingivitis. The most important findings of this study showed that the mean concentration of GCF periostin in people with chronic periodontitis significantly decreased compared to people with gingivitis and also compared to healthy people, while no significant difference was observed between the two groups with gingivitis and healthy people.

Periostin secreted from fibroblasts is found in various tissues such as serum, saliva and also GCF, and since the activity of periodontal disease can be diagnosed through GCF compounds, therefore, using the markers help to diagnose and prevent this disease [25, 26].

In the comparison between chronic periodontitis group and healthy people, the results indicated that in people with chronic periodontitis, GCF periostin concentration is significantly lower than healthy people.

In the study of Aral C et al. [26], they examined the level of periostin in GCF and saliva in non-smoker patients with chronic and aggressive periodontitis. Their results showed that the level of periostin was significantly lower in chronic and invasive periodontitis patients than in healthy individuals, which was consistent with the results of our meta-analysis. Also, the periostin level of aggressive periodontitis was significantly lower than that of chronic periodontitis.

In the study conducted by Arslan R et al. [27], the authors reported that the level of periostin in the GCF is significantly higher in people with periodontitis. Also, the level of periostin in the GCF of people with gingivitis was lower than the periodontitis group, but significantly higher than that of healthy people. The results of this study are inconsistent with the data obtained from our meta-analysis. The authors concluded that periostin increases due to the effort in tissue repair and regeneration during periodontal disease.

In Balli U et al. study [19], in addition to the concentration of periostin in the GCF, they investigated the total amount of periostin in the GCF. The results indicated that the total amount of periostin in the GCF of chronic periodontitis and gingivitis patients is significantly lower than that of healthy individuals. Also, the total amount of periostin in the GCF of people with chronic periodontitis is lower than that of people with gingivitis.

In another study that was not included in our quantitative analysis, Morsey SM et al. [28] examined the effect of non-surgical periodontal treatment on periosteum levels in patients with type 2 diabetes. In that study, there were 36 participants in three groups, including 12 people who were periodontally healthy, 12 periodontitis patients, and 12 participants with periodontitis and type 2 diabetes. The results of their study indicated that the amount of periostin in the GCF decreases in the condition of periodontitis, which was consistent with our meta-analysis.

The comparison between the gingivitis group and healthy individuals, there was no significant difference in the level of periostin in the GCF. Among the three included studies in this analysis, only the study of Akman C [18] has the similar results in compared to our meta-analysis. In the studies of Balli U [19] and Sophia K [11], the amount of periostin in the GCF of the gingivitis group is significantly lower than that of the healthy group. Therefore, more studies with a larger sample size are needed to draw accurate conclusions in this case.

The results of this meta-analysis revealed that the mean concentration of GCF periostin in people with chronic periodontitis is lower than people with gingivitis and healthy people. In addition to periodontitis, other risk factors and diseases are also effective in changing the level of periostin in saliva, GCF, serum and urine, which could not be controlled in this study. Considering that this study was a meta-analysis and the reported results in the primary studies has summarized, and in those primary studies, these risk factors and diseases were not addressed separately, the results of these cases were not adjusted in the analyses, therefore, it is recommended to interpret and generalize the results with more caution. For example, it has been shown in previous studies that asthma is associated with an increase in serum periostin levels in children [29] and in a meta-analysis study, it has been reported that periostin has moderate accuracy in diagnosing asthma [30]. In addition to these cases, in previous studies, the relationship between periostin level and diabetic retinopathy [31], renal injury [32], coronary heart disease [23], smoking [33], etc. has been shown while it was not possible to adjust these variables in the current study and the presence of people with this diseases and risk factors among the study groups can have an impact on the estimation.

Considering that the results of our study indicate that the amount of periostin in the GCF decreases in inflammatory conditions, it can be concluded that periostin plays an important role in the protection of periodontal ligament cells. Reducing the amount of periostin directly reduces the repair and formation of periodontal tissue. Also, taking into account that sampling from the GCF is a minimally invasive method, by sampling and measuring the concentration of periostin, we will obtain useful information for the early detection of periodontal disease in order to carry out timely and effective treatment.

This study had strengths and weaknesses. One of the strengths of this study is that this study, for the first time, compared the mean concentration of periostin in people with chronic periodontitis with healthy people and people with gingivitis. The limitations of this study include the following: first, in some studies, the desired numerical values are not reported and the results are reported as graphs, which means that there was no ability to extract the mean and standard deviation, and despite the correspondence with the author of the article, some of them were not included in the meta-analysis. Second, the small number of included articles in some analyzes was another limitation of this study, and for better conclusions, it is recommended to conduct more related studies.

## Conclusions

The mean concentration of GCF periostin in people with chronic periodontitis significantly decreased compared to people with gingivitis and also compared to healthy people, while no significant difference was observed between the two groups with gingivitis and healthy people. Therefore, this marker may be used as a diagnostic criterion for the disease, which requires further studies.

## Data Availability

All data generated or analyzed during this study are included in the article.

## Abbreviations

GCF: Gingival crevicular fluid
SMD: Standardized mean difference
CI: Confidence Interval
MeSH: Medical Subject Headings
TW: Text word
PRISMA: Preferred Reporting Items for Systematic Review and Meta-Analysis
PPD: Periodontal pocket depth
CAL: Clinical attachment loss
